# Short-Term Effects of Meteorological Factors and Air Pollutants on Hand, Foot and Mouth Disease among Children in Shenzhen, China, 2009–2017

**DOI:** 10.3390/ijerph16193639

**Published:** 2019-09-27

**Authors:** Siyu Yan, Lan Wei, Yanran Duan, Hongyan Li, Yi Liao, Qiuying Lv, Fang Zhu, Zhihui Wang, Wanrong Lu, Ping Yin, Jinquan Cheng, Hongwei Jiang

**Affiliations:** 1 Department of Epidemiology and Biostatistics, School of Public Health, Tongji Medical College, Huazhong University of Science and Technology, 13 Hangkong Rd, Wuhan 430030, China; 15927071586@163.com (S.Y.); dyran0116@163.com (Y.D.); hongyanli@hust.edu.cn (H.L.); zhihuiwang_hust@163.com (Z.W.); wanronglu1129@126.com (W.L.); 2 Shenzhen Center for Disease Control and Prevention, 8 Longyuan Rd, Shenzhen 518055, China; weilan@szcdc.net (L.W.); ly_jht@126.com (Y.L.); sandylv@gmail.com (Q.L.); 13714247163@163.com (F.Z.)

**Keywords:** hand, foot, and mouth disease, climatic effect, air pollution, distributed lag nonlinear model, subgroup analysis

## Abstract

Background: A few studies have explored the association between meteorological factors and hand, foot, and mouth disease (HFMD) with inconsistent results. Besides, studies about the effects of air pollutants on HFMD are very limited. Methods: Daily HFMD cases among children aged 0–14 years in Shenzhen were collected from 2009 to 2017. A distributed lag nonlinear model (DLNM) model was fitted to simultaneously assess the nonlinear and lagged effects of meteorological factors and air pollutants on HFMD incidence, and to further examine the differences of the effect across different subgroups stratified by gender, age and childcare patterns. Results: The cumulative relative risk (cRR) (median as reference) of HFMD rose with the increase of daily temperature and leveled off at about 30 °C (cRR: 1.40, 95%CI: 1.29, 1.51). There was a facilitating effect on HFMD when relative humidity was 46.0% to 88.8% (cRR at 95th percentile: 1.18, 95%CI: 1.11, 1.27). Short daily sunshine duration (5th vs. 50th) promoted HFMD (cRR: 1.07, 95%CI: 1.02, 1.11). The positive correlation between rainfall and HFMD reversed when it exceeded 78.3 mm (cRR: 1.41, 95% CI: 1.22, 1.63). Ozone suppressed HFMD when it exceeded 104 µg /m3 (cRR at 99th percentile: 0.85, 95%CI: 0.76, 0.94). NO_2_ promoted HFMD among infants and the cRR peaked at lag 9 day (cRR: 1.47, 95%CI: 1.02, 2.13) (99th vs. 50th). Besides, children aged below one year, males and scattered children were more vulnerable to high temperature, high relative humidity, and short sunshine duration. Conclusions: Temperature, relative humidity, sunshine duration, rainfall, ozone and NO_2_ were significantly associated with HFMD, and such effects varied with gender age and childcare patterns. These findings highlight the need for more prevention effort to the vulnerable populations and may be helpful for developing an early environment-based warning system for HFMD.

## 1. Introduction

Hand, foot, and mouth disease (HFMD) is a common infectious disease that mainly affects young children. It is usually characterized by fever, painful sores in the mouth, and a rash with blisters on the hands, feet, and buttocks. HFMD is caused by a group of enteroviruses, with Enterovirus A71 (EV-A71) and Coxsackievirus A16 (CV-A16) being responsible for the majority of these cases [1]. Enteroviruses are transmitted through contact with the mucus produced when an infected individual coughs or sneezes, and contact with contaminated surfaces and the feces of an infected person. Although HFMD is a self-limiting illness, a small proportion of patients particularly those infected with EV-A71 can develop severe complications that can be fatal, such as meningitis or encephalitis. Currently, there are no specific antiviral treatments for HFMD. Although two inactivated monovalent EV-A71 vaccines were licensed in China in 2015, the protective efficacy, safety, and affordability of these vaccines at the population level remains unknown, and the efficacy for HFMD caused by other viruses are unsure [2,3].

HFMD was first reported in New Zealand in 1957, and the main pathogen—EV-A71 was first identified in 1969 in California [4]. Large outbreaks of EV-A71 caused HFMD have been reported mostly in children in East and Southeast Asia as well as mainland China since 1997 [5,6,7,8,9]. HFMD was reported to be the infectious disease with the highest yearly incidence (114.48 per 100,000) and one of the five infectious diseases with high case-fatality ratio among children in mainland China between 2004 and 2013 [10]. Therefore, it is essential to identify the risk factors of HFMD and establish a targeted early warning system for future control of HFMD epidemic and reduction of disease burden.

In the context of global climate change, increasing concerns have been raised over its impact on human health. As temperatures increase, epidemic viral diseases such as HFMD may develop longer and more intense transmission seasons [11]. The seasonality of HFMD incidence also indicates a potential role of meteorological factors [12]. A few studies have explored the association between meteorological factors and HFMD with inconsistent findings. There were mainly two patterns of the effect of temperature: Some studies supported an approximate inverted V-type effect [13,14,15], while some others suggested a one-way positive effect [16,17]. A linearly positive effect as well as a non-linear effect of relative humidity on HFMD was both reported [17,18,19]. Similarly, both positive and negative effect of rainfall and sunshine duration were found [13,17,20,21,22].

It was well documented that air pollutants were harmful to non-communicable diseases [23,24,25,26]. Besides, their impacts on infectious diseases such as measles, tuberculosis and diarrhea were also reported [27,28,29]. Studies on the effects of air pollutants on HFMD just emerged in recent years and were very limited. A previous Chinese study found there was no relationship between PM_10_ and HFMD [30], whereas another study reported a positive effect of PM_10_ on HFMD in female children [31]. A recent study in China showed that low PM_2.5_ and high ozone exerted a certain protective effect on HFMD incidence [32].

Shenzhen City was taken as our research area due to its high morbidity rate of HFMD. Shenzhen owned the largest number of HFMD cases among the reported 143 cities in mainland China in the period from 2009 to 2014 [14]. Shenzhen is located in the southeast coast of China, adjacent to Hong Kong, with low latitude and a typical subtropical monsoon climate. Besides, Shenzhen is the first Special Economic Zone established in China, as well as the window of China’s reform and opening up. Based on the surveillance data of nearly a decade on HFMD, this study aimed to examine the effects of meteorological factors and air pollutants on the occurrence of HFMD by different lags, and to further examine differences in these effects across different subgroups.

## 2. Methods

### 2.1. Data Sources

Daily cases of HFMD from 2009 to 2017 were obtained from Shenzhen Center for Disease Control and Prevention. HFMD was classified as a Class C legal infectious disease of the National Surveillance System on 2 May 2008. It is required that each case should be reported to the National Notifiable Disease Reported System within 24 h after diagnosis. Considering over 99% of HFMD cases reported in the system were occurred among children under 14 years old, we excluded those aged above 14 from the analysis.

Daily meteorological data (air pressure, temperature, relative humidity, rainfall, wind speed, sunshine duration) from 2009 to 2017 were obtained from Shenzhen Meteorological Service Center. Daily level of air pollutants (SO_2_, NO_2_, CO, O_3_, PM_10_ and PM_2.5_) was recorded using the average of measurements collected from seven (eleven since 2017) state-controlled monitoring stations. All the measurements were 1-hour average, except for the measurements of O_3_ in 2017, which were 8-hour average. To maintain consistency, the data of O_3_ measured in 2017 was not included in our analysis. Because PM_2.5_ was first incorporated into air quality standards in May 2012 in China, our analysis on PM_2.5_ was based on the data after 2012.

In this study, daily scaled data was used for all analysis, as literature suggested that it might be more reasonable to use the daily scale rather than weekly especially for those diseases with shorter incubation period like HFMD [33].

### 2.2. Statistical Analysis

A distributed lag nonlinear model (DLNM) was developed in order to simultaneously assess the nonlinear and lagged effects of meteorological factors and air pollutants on HFMD incidence [34]. Allowing for over-dispersion, a quasi-Poisson regression was applied base on DLNM. The basic model is described as follows:

(1) $$Y_{t}\sim Poisson\left(\mu_{t}\right)$$

(2) $$log\left(\mu_{t}\right)=\alpha +cb\left(\mathrm{var}\right)+ns\left(\mathrm{time},\mathrm{df}=8/\mathrm{year}\right)+\beta DOW_{t}+\gamma Holiday_{t}+\tau_{t}$$

where *Y_t_* refers to the counts of HFMD cases occurred on day *t*; *α* is the intercept; *var* is meteorological variable or air pollutant; *cb* refers to the cross-basis function, combining functions for both exposure and lag dimensions. In the *cb* functions with two degrees of freedom (*df*) and one *lag*, i.e., *cb* (factor, *df*_1_, *lag*, *df*_2_), natural cubic spline with *df*_1_ ranged from 2 to 6 was used to capture the nonlinear exposure–response curve of meteorological factors and air pollutants; a maximum *lag* of 14 d was applied to reflect the incubation period of 3–10 days for HFMD, and natural cubic spline with *df*_2_ ranged from 2 to 4 was employed to represent the lag–response curve of these factors; the criterion of minimizing quasi-Akaike information criterion (QAIC) was adopted to optimize the *cb* function, as reported in previous studies (seen in Table S1) [35,36]. The long-term trend and seasonality were depicted using natural cubic spline function of time, denoted by *ns* (time). This is because natural cubic spline for time was less biased than penalize spline [36]. The eight degrees of freedom per year for *ns* (time) was determined using the QAIC and sum of partial autocorrelation coefficients (PACF), as the selection of degree of freedom for *ns* (time) described in previous literature (seen in Table S2 and Figure S1) [14]. *DOW_t_* is day of the week on day *t*; *Holiday_t_* is a binary variable for adjusting the potential effect of public holidays including winter/summer school holidays; *τ_t_* refers to the autoregressive terms of daily HFMD counts on the logarithmic scale at *lag* 1 and *lag* 2 to control for the autocorrelations occurring in cases of infectious disease (seen in Table S3 and Figure S2) [37]; *β* and *γ* are the coefficients of the corresponding terms. In order to examine the influence of extreme weather and pollution conditions, median of every factor was adopted as reference in calculation of the cumulative relative risk (cRR), which reflects the relative risk to its average level.

In order to obtain the most concise model, we borrowed ideas from stepwise regression to integrate six steps into a variable screening approach based on the following criteria: (1) Biological rationality based on the laboratory evidence or other literature; (2) statistical significance on outcomes; (3) avoiding concurvity; and (4) simplicity of the model. First, univariate models were used to exclude those statistically insignificant factors (seen in Figure S3). Second, spearman rank correlation test was taken for assessing concurvity between factors (seen in Table S4). Third, a stepwise method for variable screening with a cutoff probability of 0.05 for adding variables and that of 0.05 for removing variables was adopted to select those statistically significant factors. Fourth, three statistical indices were administered to analyze the interactions of those selected factors, including relative excess risk due to interaction, attributable proportion, and synergy index [38]. Fifth, to reduce the complexity of DLNM, QAIC was introduced to select plausible meteorological factors (seen in Table S5). Different choices of function for meteorological covariate were compared, which varied in different studies, including natural cubic spline of the current value, exponential moving average and cross-basis function (seen in Table S6). Sixth, the results from the above mentioned stepwise selection were combined with professional knowledge to select those plausible air pollutants. This variable screening procedure helped us select two meteorological factors (air temperature and relative humidity) and two air pollutants (NO_2_ and O_3_) for creating the most concise model. No first-order interactions between these factors were brought into regression models due to its statistical insignificance. Additionally, the other factors (e.g., sunshine duration and rainfall) were introduced one by one to this model as the QAIC values of several statistical models were close to the minimal QAIC of the most concise model. Besides, we conducted the sensitivity analysis by changing *df* (7–9) of controlling long-term trends and seasonality and changing the maximum lag days to 21 days for meteorological variables and air pollutants (seen in Figure S4). Further, we also stratified the analysis by gender, age and childcare patterns to explore the consistency of inter-layer results and identify sensitive populations. R software version 3.4.4 (R Foundation for Statistical Computing, Vienna, Austria) and SAS V9.4 (Sai Shi Software, Cary, NC, USA) were used for data analysis. Statistical significance was determined by the 95% confidence interval.

## 3. Results

### 3.1. Descriptive Analysis

Between 1 January 2009 and 31 December 2017, there were a total of 357,238 HFMD cases occurred in children aged 0–14 years in Shenzhen, with 75% of cases occurred among children aged 3 years or below. There were more male cases with a male-to-female ratio of 1.56:1. The average number of daily childhood HFMD cases was 106.6 (range: 0–860). Nearly 97% of the children could be categorized directly as scattered children (children living at home) or nursery children. Moreover, the descriptive summary of daily meteorological variables and air pollutants were also provided in Table 1.

**Figure 1:**
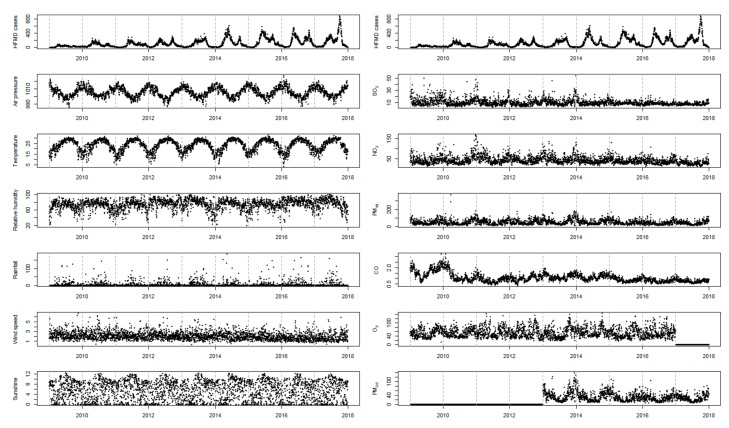
Daily distribution of children HFMD cases, meteorological variables and air pollutants in Shenzhen, China, 2009–2017 (the data of O_3_ in 2017 and PM_2.5_ before 2013 were missing).

Figure 1 showed the time-series distributions of daily HFMD cases, meteorological variables and air pollutants from 1 January 2009 to 31 December 2017 in Shenzhen (excepting for O_3_ and PM_2.5_). There were some seasonal pattern and long-term trend for these variables in general. For instance, there was an obvious seasonality for the HFMD occurrence with two epidemic peaks every year as well as an annual growth trend.

### 3.2. Associations between Meteorological Variables and HFMD Incidence

**Figure 2:**
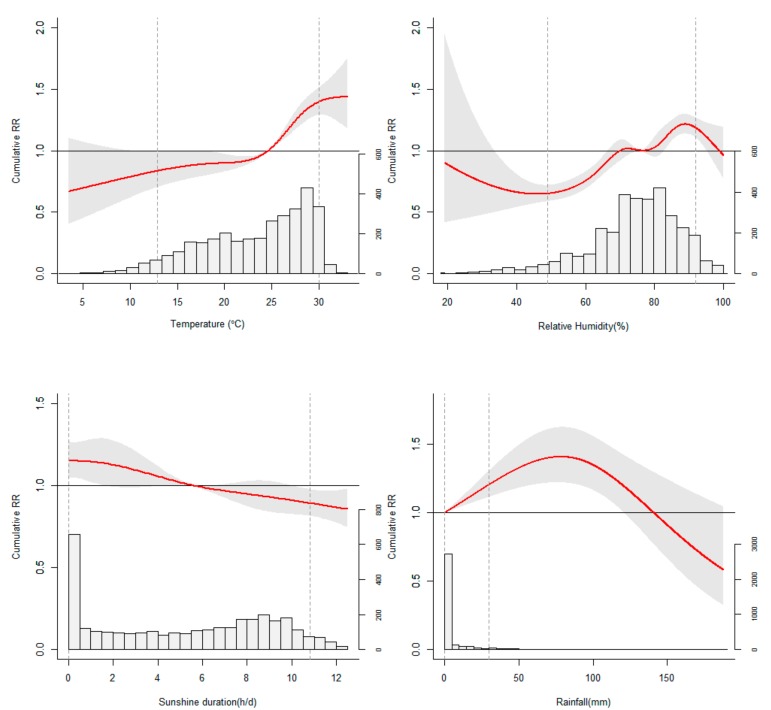
The histograms of meteorological variables and their corresponding estimated overall cumulative association with HFMD occurrence over 14 days. Dotted lines mean 5th percentile (P5) and 95th percentile (P95) respectively.

Figure 2 showed the cumulative associations between meteorological variables and HFMD occurrence over 14 days in the bi-meteorological variable models. The cRR of relative humidity on HFMD grew gradually from a minimum of 0.65 (95% CI: 0.57, 0.73) to a maximum of 1.21 (95% CI: 1.14, 1.30) within its range from 46.0% to 88.8%. Outside this range, however, the cRR declined with the increase of relative humidity. Sunshine duration showed a nearly linear negative effect, and the cRR at 5th and 95th percentiles were 1.15 (95% CI: 1.05, 1.27) and 0.89 (95% CI: 0.81, 0.98) respectively. The effect of rainfall showed an inverted V-shape peaking at 78.3 mm (the corresponding maximum cRR: 1.41, 95% CI: 1.22, 1.63) and turned insignificant above 120.6 mm. The cumulative effects of meteorological variables at 5th and 95th percentiles were displayed specifically in Table 2.

**Figure 3:**
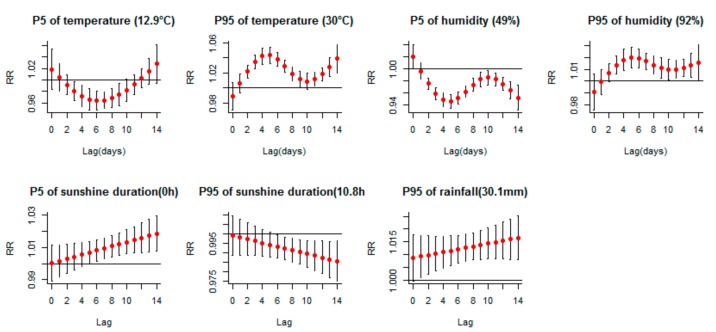
Lag-response curves for P5, P95 of meteorological variables on HFMD occurrence (5th percentile of rainfall was 0 mm, the same with the reference value, so the effect of 5th percentile of rainfall was not shown). The red points are the relative risks (medians as references), and the black bars are 95% CIs. Shenzhen 2009–2017.

The lag–response curves in Figure 3 showed that cold effect (5th percentile of temperature vs. median) turned significant at *lag* 3, peaked at *lag* 6 and lasted until *lag* 9. While hot effect (95th percentile of temperature vs. median) turned significant at *lag* 2 and peaked at *lag* 5. Dry effect (5th percentile of relative humidity vs. median) turned significant at lag 2, while wet effect (95th percentile vs. median) turned significant at lag 3. They peaked both at lag 5. Risk of short sunshine duration (5th vs. median) and heavy rainfall (95th vs. median) continued to increase as lag days extended. A sensitivity analysis of the *lag*–response curves of air temperature and relative humidity with a max lag of 30 days were conducted and illustrated in Supplementary Materials Figure S5.

### 3.3. Associations between Air Pollutants and HFMD Incidence

**Figure 4:**
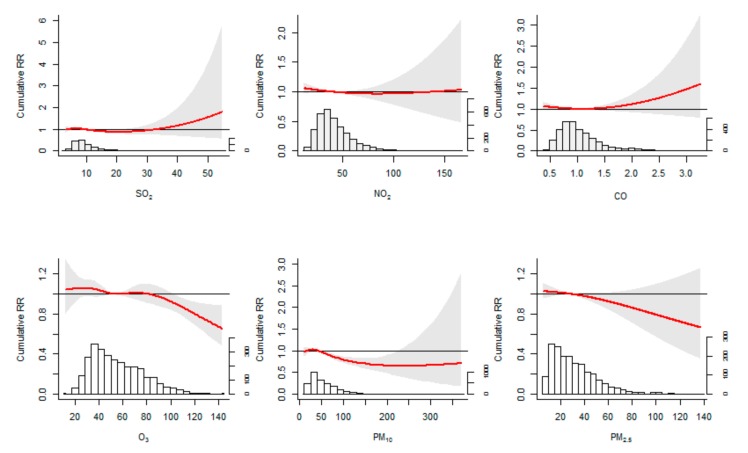
The estimated overall cumulative association between air pollutants and HFMD occurrence over 14 days with their distributions, using a natural cubic spline distributed lag nonlinear model adjusted by temperature and relative humidity. The red lines are the cumulative relative risks (medians as references), and the gray regions are 95% CIs. Shenzhen 2009–2017.

The overall cumulative associations over 14 days between air pollutants and HFMD occurrence were showed in Figure 4. Among the various air pollutants examined in this study, the effect of O_3_ was significant. When the O_3_ concentration was greater than 104 µg/m^3^ (corresponding cRR: 0.90, 95% CI: 0.82, 0.99), the risk of HFMD decreased significantly. The cRR at 99th percentile of O_3_ were 0.85 (95% CI: 0.76, 0.94). The effect of NO_2_ on HFMD among children aged below 14 years appeared to be non-significant. A positive effect of SO_2_ and CO was observed but insignificant, as well as the negative effect of PM_2.5_ and PM_10_.

The results were similar when the maximum lag was increased to 21d and the *df* for controlling long-term trends and seasonality varied from 7 to 9 (seen in Figure S4).

### 3.4. Subgroup Analysis

**Figure 5:**
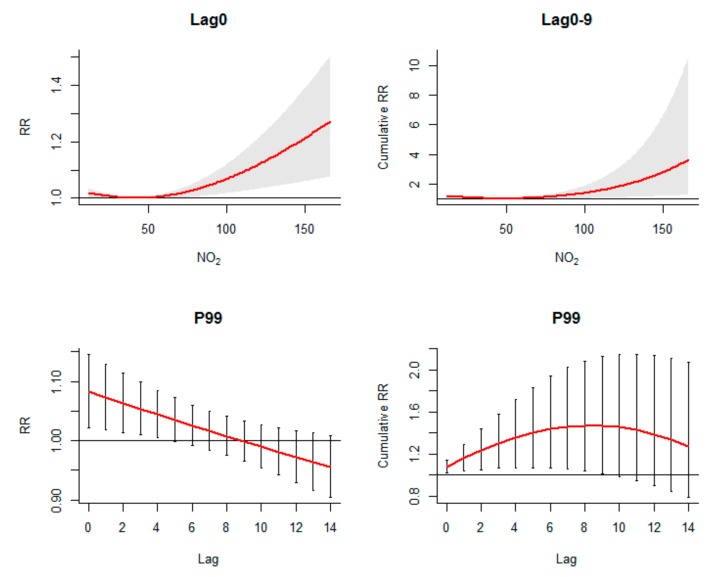
The exposure–response curves of NO_2_ over lag 0 and lag 0–9; lag–response curves for 99th percentile of NO_2_ (106.1 µg/m^3^), calculating single-lag effect and cumulative effect separately.

The results of subgroup analysis were displayed in Table 2. A key finding was that NO_2_ increased the risk of HFMD incidence of children aged 0~1 year. The single-lag effect of NO_2_ peaked at lag 0, and the RR value was 1.08 (95% CI: 1.02, 1.15). The cumulative effect of NO_2_ peaked at lag 9 and the cRR at 99th percentile was 1.47 (95% CI: 1.02, 2.13) (Figure 5). Besides, the protective cold effect was stronger in females than males. Children aged below one year were most sensitive to both cold effect and hot effect. Compared with nursery children, the hot effect was stronger in scattered children. The dry and wet effects were similar in these subgroups, except that children aged below one year were more sensitive than other age groups. The adverse effect of short sunshine duration on HFMD was significant except for children aged 3–14 years and nursery children; the protective effect of long sunshine duration was significant except for females, infants and scattered children. The impact of 95th percentile of rainfall on HFMD incidence was highest in children aged below one year, and was similar in different gender and childcare pattern. The protective effect of O_3_ was insignificant in children aged below one year and was stronger in nursery children compared with scattered children.

## 4. Discussion

In this study, we found that temperature, relative humidity, sunshine duration, rainfall, O_3_ and NO_2_ were significantly associated with the incidence of childhood HFMD, and the associations varied with gender age and childcare patterns.

We found the risk of HFMD incidence rose with the increase of daily temperature and leveled off at about 30 °C. Our study confirmed the facilitating effect of temperature on HFMD, which was consistent with studies in China and other countries [39]. Firstly, the reason may be that the infectivity of enterovirus increases with temperature over specific range (Hagiwara et al. 1983). Secondly, when the temperature is moderate, children take more outdoor activity and may have higher chance of exposure to infected individuals and contaminated surfaces [40,41]. For relative humidity, when it was between 46.0% and 88.8%, the risk of HFMD incidence rose with the increase of relative humidity. The two thresholds were similar to that reported in another study in Shenzhen [17]. The extreme high levels of air temperature and relative humidity could increase the delayed incidence risk of HFMD, while their extreme low levels could decrease it. We also found an inverted V-shape effect of rainfall with the turning point of 78.3 mm, similar to a study in Guangdong with a lower peaking point (25 mm) [19]. In contrast to our finding, a study in Hefei suggested a positive effect of extreme precipitation (≥90th precipitation) on HFMD [20]. Firstly, the inconsistent finding might be due to the difference in measurement of rainfall. They treated rainfall as a categorical variable, but we incorporated it as a continuous variable into the model in order to depict it more specifically. Secondly, the average rainfall in Shenzhen was heavier than that in Hefei, thus our study could explore the effect of extreme rainfall on HFMD. A laboratory research showed no infectivity of Enterovirus B was recovered from dried soil and the rate of virus inactivation increased at low soil moisture levels [42]. An epidemiological study in Korea suggested that person-to-person contact and contaminated water could be the principal modes of transmission of HFMD [43]. Enteroviruses have been isolated from various types of water, including groundwater, treated sewage, marine water, and drinking water. We presume the positive effects of relative humidity and rainfall within a certain range could result from providing a suitable environment for activation and infectivity of enterovirus and giving more opportunities of water as a medium of communication. When relative humidity was above 88.8%, the decrease of HFMD incidence risk may be attributed to less physical outdoor activities of children due to discomfort and increased fatigue in extreme relative humidity and correspondingly less contact with infected person or contaminated surfaces, or slower excretion of feces with virus [44]. Similarly, the declines of risk under heavier rainfall (above 78.3 mm) could be attributed to less outdoor activities of host and susceptible person [40]. A nearly linear negative effect of sunshine duration on HFMD incidence was observed in our study, consistent with a study in Shanghai [13]. The reason may be the inactivation of virus caused by ultraviolet and evaporation of water in the environment under long sunshine duration.

As for the air pollutants, there were some noteworthy findings. O_3_ showed a significant protective effect on HFMD when it was greater than 104 µg/m^3^. So far, only one study explored association between O_3_ and HFMD, and it suggested the effect is possibly attributed to its inhibitory effect on the ability of enterovirus to survive or replicate in the external environment [32]. Ozone has been shown to possess broad-spectrum antimicrobial and antiviral activity [45]. Lin et al. demonstrated the potential of ozone for EV71 inactivation and found the ozone exposure of uninfected cells could stimulate cytokine production, which may help to suppress the virus replication upon EV71 infection [46,47]. Based on this finding, we speculated a possibility of ozone therapy for HFMD, though no relevant clinical practice has yet been seen. Future studies are required to validate this speculate. What’s more, for children aged 0~1 year, NO_2_ increased the risk of HFMD incidence. This is the first epidemiological study on the association between short-term exposure of NO_2_ and HFMD cases to our knowledge. Vehicle exhaust is one of the main sources of urban NO_2_ and the rapid economic growth in Shenzhen boosted the car ownership, thus NO_2_ pollution in Shenzhen is relatively serious [48]. Although no laboratory studies have been found on the direct effects of NO_2_ on enteroviruses, a study about NO_2_ and other intestinal infectious disease indicated a positive correlation between the concentration of NO_2_ and the morbidity of rotavirus [28]. Given that the oral route also accounts for much of the exposure to air pollutants as the pollutants contaminate the food and water supply in significant amounts, NO_2_ may have direct effects on epithelial cells, cause systemic inflammation and immune activation, and modulate the intestinal microbiota [49,50]. Another laboratory study showed NO_2_ may decrease virus-specific immunity in mice and increase the inflammation of cells regardless of whether NO_2_ exposure precedes or succeeds respiratory viral infections [51]. HFMD can also be spread by droplets, thus we suspect NO_2_ may affect HFMD in the same way as respiratory viral infections, increasing the risk of HFMD incidence by affecting immunity and inflammation and weakening the body’s resistance to viral infection. Similarly, the significant harmful effect in children aged 0–1 year could be attributed to their relatively immature immune systems. More research is needed to confirm the association between short-term exposure of air pollutants and HFMD incidence and to explore the potential mechanism.

The subgroup analysis showed that children aged 0–1 year were most vulnerable to the adverse effect of high temperature, high relative humidity, short sunshine duration, and heavy rainfall. Infants have poor self-care ability and relatively immature immune systems, and thus are more vulnerable to these risk factors [52]. But they were not sensitive to the protective effect of O_3_. As we mentioned previously, the protective effect of ozone could be attributed to its inhibition on enterovirus in external environment. Infants are less likely to contact with contaminated surfaces due to their limited daily activity, thus being less protected by ozone. Compared with females, males had higher risk of HFMD incidence under high relative humidity, heavy rainfall, and were significantly protected under long sunshine duration. Maybe it is because males take more outdoor activities than females, which may increase their exposures to pathogen or contaminated surfaces [40]. Besides, females usually produce more vigorous cellular and more vigorous humoral immune reactions, and thus are more resistant to infections [53]. Compared with nursery children, scattered children were under a higher risk in days with high temperature and short sunshine duration, as well as a weaker protective effect of O_3_. Possible reasons may be that scattered children have poor sanitary conditions and poor hygienic practices due to the lack of supervision, thus they need more attention for the prevention effort.

There are some strengths of this study: firstly, the sample size is relatively large, with 357,238 HFMD cases among children aged below 14 years in Shenzhen since HFMD has been classified as a statutory reporting disease in 2008. Secondly, this is the first study to investigate the individual effect of various air pollutants on HFMD in Shenzhen, China. Methodologically, we have improved the model by screening variables to avoid severe concurvity, and controlling for autocorrelations caused by disease transmission. Thirdly, the analysis of effects on children was stratified by gender, age, and childcare patterns to identify susceptible populations.

Some limitations of this study warrant mention. Firstly, our study is a time series study, which is essentially an ecological design and has limitation in causal inference. Secondly, as not all infected people seek medical care, HFMD cases reported in the surveillance system may be underestimated. Thirdly, we had no access to the monitoring data on PM_2.5_ before 2013 and O_3_ after 2016. Further studies with complete data on these two air pollutants are needed. Fourthly, Shenzhen is under a relatively good air condition, thus our study fail to examine the effect of heavy air pollution on HFMD. Lastly, as an important source of variation of HFMD incidence, regional heterogeneity was excluded from this study due to the ethical prohibition on collecting personal geographical information. Daily routing analysis based on random walk model will be the focus of future research in HFMD incidence.

## 5. Conclusions

Our study provided significant evidence on the nonlinear relationship and lagged effect between daily meteorological variables and HFMD incidence, and contributed to the limited knowledge of impacts of air pollutants on HFMD incidence. Our study identified the harmful effect of NO_2_ on HFMD among children below one year for the first time. Also, we found an inverted V-shape effect of rainfall and a protective effect of ozone on HFMD. Other meteorological factors such as temperature, relative humidity, sunshine duration were also significantly associated with HFMD occurrence. Moreover, children below one year, males and scattered children were more vulnerable to high temperature, high relative humidity and short sunshine duration. These findings highlight the need for more prevention effort to the vulnerable populations and may be helpful for developing an early environment-based warning system for HFMD.

## Figures and Tables

**Table 1:** Descriptive statistics of daily children hand, foot, and mouth disease (HFMD) cases, meteorological variables and air pollutants in Shenzhen, China, 2009–2017.

Variables	*N*	Mean	Standard Deviation	Minimum	25% Quartile	Median	75% Quartile	Maximum
Daily HFMD cases								
Total (0~14y)	357,238	106.6	117.4	0.0	22.0	65.0	156.0	860.0
Children < 1y	50,657	15.4	20.2	0.0	2.0	8.0	21.0	172.0
1 ≤ Children < 3y	185,440	56.4	63.1	0.0	11.0	33.0	83.0	517.0
Children ≥ 3y	121,141	36.9	43.9	0.0	8.0	22.0	48.0	346.0
Males	217,720	66.2	72.2	0.0	14.0	40.0	100.0	523.0
Females	139,518	42.5	47.7	0.0	8.0	25.0	62.0	353.0
Scattered children	279,478	85.0	95.5	0.0	16.0	49.0	124.5	738.0
Nursery children	67,000	20.4	27.3	0.0	3.0	10.0	26.0	269.0
Meteorological variables								
Air pressure (hpa)	3287	1005.3	6.4	986.0	1000.6	1005.1	1010.2	1027.3
Temperature (°C)	3287	23.3	5.6	3.5	19.1	24.6	28.0	33.0
Relative Humidity (%)	3287	74.3	13.0	19.0	68.0	76.0	83.0	100.0
Rainfall (mm)	3287	4.9	15.2	0.0	0.0	0.0	1.0	187.8
Wind speed (m/s)	3287	2.1	0.8	0.3	1.5	2.0	2.5	6.7
Sunshine duration (h/d)	3287	5.2	3.8	0.0	1.3	5.6	8.7	12.5
Air pollutants								
SO_2_ (µg/m^3^)	3287	10.3	5.2	3.0	7.0	8.9	12.0	54.8
NO_2_ (µg/m^3^)	3287	42.3	18.3	12.0	29.7	38.3	50.5	166.1
CO (mg/m^3^)	3287	1.1	0.4	0.4	0.8	1.0	1.2	3.3
O_3_ (µg/m^3^)_2009–2016_	2922	55.5	21.7	11.8	38.3	51.7	69.9	143.3
PM_10_ (µg/m^3^)	3287	51.6	28.4	8.6	30.0	44.8	67.3	374.2
PM_2.5_ (µg/m^3^)_2013–2017_	1826	32.1	19.7	5.6	16.9	27.7	42.3	137.1

**Table 2:** Cumulative effects of meteorological variables and air pollutants on HFMD occurrence over 14 days (except for NO_2_) in total cases and different subgroups, estimated by cumulative relative risk (cRR) and 95% CI in Shenzhen, 2009–2017.

Variables	Total	Male	Female	0~1	1~3	3~14	Scattered Children	Nursery Children
Meteorological variables								
Temperature (P5)	**0.83(0.70, 0.99)**	**0.82(0.68, 0.99)**	**0.76(0.61, 0.93)**	**0.58(0.4, 0.84)**	**0.65(0.53, 0.80)**	**0.79(0.63, 0.98)**	**0.79(0.66, 0.95)**	0.90(0.70, 1.17)
Temperature (P95)	**1.40(1.29, 1.51)**	**1.46(1.34, 1.60)**	**1.46(1.33, 1.60)**	**2.03(1.77, 2.33)**	**1.65(1.50, 1.81)**	**1.31(1.16, 1.48)**	**1.48(1.36, 1.60)**	**1.25(1.08, 1.45)**
Humidity (P5)	**0.65(0.59, 0.72)**	**0.62(0.56, 0.70)**	**0.61(0.54, 0.69)**	**0.49(0.41, 0.59)**	**0.60(0.53, 0.67**)	**0.66(0.58, 0.76)**	**0.62(0.56, 0.69)**	**0.64(0.54, 0.76)**
Humidity (P95)	**1.18(1.11, 1.27)**	**1.22(1.13, 1.31)**	**1.18(1.09, 1.28)**	**1.44(1.28, 1.63)**	**1.21(1.12, 1.3)**	**1.17(1.06, 1.28)**	**1.20(1.12, 1.29)**	**1.19(1.06, 1.34)**
Sunshine duration (P5)	**1.15(1.05, 1.27)**	**1.18(1.06, 1.31)**	**1.17(1.04, 1.31)**	**1.51(1.29, 1.78)**	**1.17(1.05, 1.31)**	**1.00(0.87, 1.15)**	**1.18(1.07, 1.30)**	1.11(0.94, 1.3)
Sunshine duration (P95)	**0.89(0.81, 0.98)**	**0.88(0.80, 0.98)**	0.90(0.81, 1.01)	0.99(0.85, 1.14)	**0.88(0.80, 0.98)**	**0.74(0.64, 0.85)**	0.92(0.83, 1.00)	**0.78(0.65, 0.92)**
Rainfall (P95)	**1.21(1.12, 1.31)**	**1.25(1.14, 1.36)**	**1.23(1.12, 1.35)**	**1.45(1.27, 1.65)**	**1.25(1.14, 1.37)**	**1.36(1.21, 1.53)**	**1.23(1.13, 1.33)**	**1.26(1.09, 1.45)**
Air pollutants								
O_3_ (P99)	**0.85(0.76, 0.94)**	**0.82(0.73, 0.93)**	**0.84(0.74, 0.96)**	**0.87(0.73, 1.04)**	**0.85(0.75, 0.96)**	**0.78(0.67, 0.92)**	**0.87(0.78, 0.98)**	**0.70(0.57, 0.86)**
NO_2_ (P99), lag 0	1.02(0.99, 1.05)	1.02(0.99, 1.06)	1.02(0.98, 1.06)	**1.08(1.02, 1.15)**	1.02(0.98, 1.05)	1.01(0.97, 1.05)	1.02(0.99, 1.06)	1.01(0.96, 1.06)
NO_2_ (P99), lag 0–9	1.05(0.86, 1.28)	1.08(0.87, 1.34)	1.04(0.81, 1.33)	**1.47(1.02, 2.13)**	1.04(0.83, 1.30)	1.00(0.77, 1.30)	1.09(0.88, 1.33)	1.06(0.76, 1.48)

Bold: for *p* < 0.05.

## Supplementary Materials

The following are available online at https://www.mdpi.com/1660-4601/16/19/3639/s1. Table S1: The df of exposure and lag determined in the cb function, Table S2: The QAIC and sum of PACF for different methods, Table S3: The magnitude of the PACF plot for the first two lag days in different models, Table S4: Spearman’s correlation coefficients between meteorological variables and air pollutants in Shenzhen, China, 2009–2017, Table S5: QAIC of multi-meteorological factor models, Figure S1: QAIC and sum of PACF for different df of time splines, Table S6: Compare of the different functions of covariates, Figure S2: The ACF and PACF plot of residuals after removing long-term trends and seasonality (by the time splines with 8 df per year), Figure S3: The estimated overall cumulative association between meteorological variables and HFMD occurrence over 14 days with their distributions, using a natural cubic spline DLNM in uni-meteorological variable models. The red lines are the cumulative relative risks (medians as references), and the gray regions are 95% CIs. Shenzhen 2009–2017, Figure S4: Results of sensitivity analyses by changing the df for controlling long-term trends and seasonality from 7 to 9 and changing the maximum lag days to 21 days, showing the estimated overall cumulative effects over maxlag days (except for NO_2_ over lag0), Figure S5: Lag-response curves with a max lag of 30 for P5, P95 of air temperature and relative humidity on HFMD occurrence. The red points are the relative risks (medians as references), and the black bars are 95% CIs. Shenzhen 2009–2017.

## References

[1] Xing W., Liao Q., Viboud C., Zhang J., Sun J., Wu J.T., Chang Z., Liu F., Fang V.J., Zheng Y. (2014). Hand, foot, and mouth disease in China, 2008–2012: An epidemiological study. Lancet Infect. Dis..

[2] Takahashi S., Liao Q., van Boeckel T.P., Xing W., Sun J., Hsiao V.Y., Metcalf C.J.E., Chang Z., Liu F., Zhang J. (2016). Hand, Foot, and Mouth Disease in China: Modeling Epidemic Dynamics of Enterovirus Serotypes and Implications for Vaccination. PLoS Med..

[3] Zhu F.C., Meng F.Y., Li J.X., Li X.L., Mao Q.Y., Tao H., Zhang Y.T., Yao X., Chu K., Chen Q.H. (2013). Efficacy, safety, and immunology of an inactivated alum-adjuvant enterovirus 71 vaccine in children in China: A multicentre, randomised, double-blind, placebo-controlled, phase 3 trial. Lancet.

[4] Schmidt N.J., Lennette E.H., Ho H.H. (1974). An apparently new enterovirus isolated from patients with disease of the central nervous system. J. Infect. Dis..

[5] Ahmad K. (2000). Hand, foot, and mouth disease outbreak reported in Singapore. Lancet.

[6] Chatproedprai S., Theanboonlers A., Korkong S., Thongmee C., Wananukul S., Poovorawan Y. (2010). Clinical and molecular characterization of hand-foot-and-mouth disease in Thailand, 2008–2009. Jpn. J. Infect. Dis..

[7] Khanh T.H., Sabanathan S., Thanh T.T., Thoa L.P.K., Thuong T.C. (2012). Enterovirus 71-associated hand, foot, and mouth disease, Southern Vietnam, 2011. Emerg. Infect. Dis..

[8] Komatsu H., Shimizu Y., Takeuchi Y., Ishiko H., Takada H. (1999). Outbreak of severe neurologic involvement associated with enterovirus 71 infection. Pediatric Neurol..

[9] Wang J.R., Tsai H.P., Chen P.F., Lai Y.J., Yan J.J., Kiang D., Lin K.H., Liu C.C., Su I.J. (2000). An outbreak of enterovirus 71 infection in Taiwan, 1998. II. Laboratory diagnosis and genetic analysis. J. Clin. Virol. Off. Publ. Pan Am. Soc. Clin. Virol..

[10] Yang S., Wu J., Ding C., Cui Y., Zhou Y., Li Y., Deng M., Wang C., Xu K., Ren J. (2017). Epidemiological features of and changes in incidence of infectious diseases in China in the first decade after the SARS outbreak: An observational trend study. Lancet Infect. Dis..

[11] Kaffenberger B.H., Shetlar D., Norton S.A., Rosenbach M. (2017). The effect of climate change on skin disease in North America. J. Am. Acad. Derm..

[12] Chen K.T., Chang H.L., Wang S.T., Cheng Y.T., Yang J.Y. (2007). Epidemiologic features of hand-foot-mouth disease and herpangina caused by enterovirus 71 in Taiwan, 1998–2005. Pediatrics.

[13] Qi H., Chen Y., Xu D., Su H., Zhan L., Xu Z., Huang Y., He Q., Hu Y., Lynn H. (2018). Impact of meteorological factors on the incidence of childhood hand, foot, and mouth disease (HFMD) analyzed by DLNMs-based time series approach. Infect. Dis. Poverty.

[14] Xiao X., Gasparrini A., Huang J., Liao Q., Liu F., Yin F., Yu H., Li X. (2017). The exposure-response relationship between temperature and childhood hand, foot and mouth disease: A multicity study from mainland China. Environ. Int..

[15] Zhu L., Wang X., Guo Y., Xu J., Xue F., Liu Y. (2016). Assessment of temperature effect on childhood hand, foot and mouth disease incidence (0–5 years) and associated effect modifiers: A 17 cities study in Shandong Province, China, 2007–2012. Sci. Total Environ..

[16] Urashima M., Shindo N., Okabe N. (2003). Seasonal models of herpangina and hand-foot-mouth disease to simulate annual fluctuations in urban warming in Tokyo. Jpn. J. Infect. Dis..

[17] Zhang Z., Xie X., Chen X., Li Y., Lu Y., Mei S., Liao Y., Lin H. (2016). Short-term effects of meteorological factors on hand, foot and mouth disease among children in Shenzhen, China: Non-linearity, threshold and interaction. Sci. Total Environ..

[18] Huang Y., Deng T., Yu S., Gu J., Huang C., Xiao G., Hao Y. (2013). Effect of meteorological variables on the incidence of hand, foot, and mouth disease in children: A time-series analysis in Guangzhou, China. BMC Infect. Dis..

[19] Zhang W., Du Z., Zhang D., Yu S., Hao Y. (2016). Boosted regression tree model-based assessment of the impacts of meteorological drivers of hand, foot and mouth disease in Guangdong, China. Sci. Total Environ..

[20] Cheng J., Wu J., Xu Z., Zhu R., Wang X., Li K., Wen L., Yang H., Su H. (2014). Associations between extreme precipitation and childhood hand, foot and mouth disease in urban and rural areas in Hefei, China. Sci. Total Environ..

[21] Nguyen H.X., Chu C., Nguyen H.L.T., Nguyen H.T., Do C.M., Rutherford S., Phung D. (2017). Temporal and spatial analysis of hand, foot, and mouth disease in relation to climate factors: A study in the Mekong Delta region, Vietnam. Sci. Total Environ..

[22] Phung D., Nguyen H.X., Nguyen H.L.T., Do C.M., Dai Tran Q., Chu C. (2018). Spatiotemporal variation of hand-foot-mouth disease in relation to socioecological factors: A multiple-province analysis in Vietnam. Sci. Total Environ..

[23] Guan W.J., Zheng X.Y., Chung K.F., Zhong N.S. (2016). Impact of air pollution on the burden of chronic respiratory diseases in China: Time for urgent action. Lancet.

[24] Guarnieri M., Balmes J.R. (2014). Outdoor air pollution and asthma. Lancet.

[25] Shah A.S., Langrish J.P., Nair H., McAllister D.A., Hunter A.L., Donaldson K., Newby D.E., Mills N.L. (2013). Global association of air pollution and heart failure: A systematic review and meta-analysis. Lancet.

[26] Simkhovich B.Z., Kleinman M.T., Kloner R.A. (2008). Air pollution and cardiovascular injury epidemiology, toxicology, and mechanisms. J. Am. Coll. Cardiol..

[27] Chen G., Zhang W., Li S., Williams G., Liu C., Morgan G.G., Jaakkola J.J., Guo Y. (2017). Is short-term exposure to ambient fine particles associated with measles incidence in China? A multi-city study. Environ. Res..

[28] Ye Q., Fu J.F., Mao J.H., Shen H.Q., Chen X.J., Shao W.X., Shang S.Q., Wu Y.F. (2016). Haze is an important medium for the spread of rotavirus. Environ. Pollut..

[29] Zhu S., Xia L., Wu J., Chen S., Chen F., Zeng F., Chen X., Chen C., Xia Y., Zhao X. (2018). Ambient air pollutants are associated with newly diagnosed tuberculosis: A time-series study in Chengdu, China. Sci. Total Environ..

[30] Huang R., Bian G., He T., Chen L., Xu G. (2016). Effects of Meteorological Parameters and PM10 on the Incidence of Hand, Foot, and Mouth Disease in Children in China. Int. J. Environ. Res. Public Health.

[31] Huang R., Ning H., He T., Bian G., Hu J., Xu G. (2018). Impact of PM10 and meteorological factors on the incidence of hand, foot, and mouth disease in female children in Ningbo, China: A spatiotemporal and time-series study. Environ. Sci. Pollut. Res..

[32] Yu G., Li Y., Cai J., Yu D., Tang J., Zhai W., Wei Y., Chen S., Chen Q., Qin J. (2019). Short-term effects of meteorological factors and air pollution on childhood hand-foot-mouth disease in Guilin, China. Sci. Total Environ..

[33] Cheng Q., Bai L., Zhang Y., Zhang H., Wang S., Xie M., Zhao D., Su H. (2018). Ambient temperature, humidity and hand, foot, and mouth disease: A systematic review and meta-analysis. Sci. Total Environ..

[34] Gasparrini A., Armstrong B., Kenward M.G. (2010). Distributed lag non-linear models. Stat. Med..

[35] Armstrong B. (2006). Models for the relationship between ambient temperature and daily mortality. Epidemiology.

[36] Peng R.D., Dominici F., Louis T.A. (2006). Model choice in time series studies of air pollution and mortality. J. R. Stat. Soc. Ser. A Stat. Soc..

[37] Imai C., Armstrong B., Chalabi Z., Mangtani P., Hashizume M. (2015). Time series regression model for infectious disease and weather. Environ. Res..

[38] Andersson T., Alfredsson L., Källberg H., Zdravkovic S., Ahlbom A. (2005). Calculating measures of biological interaction. Eur. J. Epidemiol..

[39] Zhao Q., Li S., Cao W., Liu D.L., Qian Q., Ren H., Ding F., Williams G., Huxley R., Zhang W. (2018). Modeling the Present and Future Incidence of Pediatric Hand, Foot, and Mouth Disease Associated with Ambient Temperature in Mainland China. Environ. Health Perspect..

[40] Duncan J.S., Hopkins W.G., Schofield G., Duncan E.K. (2008). Effects of weather on pedometer-determined physical activity in children. Med. Sci. Sports Exerc..

[41] Edwards N.M., Myer G.D., Kalkwarf H.J., Woo J.G., Khoury P.R., Hewett T.E., Daniels S.R. (2015). Outdoor Temperature, Precipitation, and Wind Speed Affect Physical Activity Levels in Children: A Longitudinal Cohort Study. J. Phys. Act. Health..

[42] Yeager J.G., O’Brien R.T. (1979). Enterovirus inactivation in soil. Appl. Environ. Microbiol..

[43] Park S.K., Park B., Ki M., Kim H., Lee K., Jung C., Sohn Y.M., Choi S.M., Kim D.K., Lee D.S. (2010). Transmission of Seasonal Outbreak of Childhood Enteroviral Aseptic Meningitis and Hand-foot-mouth Disease. J. Korean Med. Sci..

[44] Remmers T., Thijs C., Timperio A., Salmon J.O., Veitch J., Kremers S.P., Ridgers N.D. (2017). Daily Weather and Children’s Physical Activity Patterns. Med. Sci. Sports Exerc..

[45] Akey D.H., Walton T.E. (1985). Liquid-phase study of ozone inactivation of Venezuelan equine encephalomyelitis virus. Appl. Environ. Microbiol..

[46] Lin Y.C., Juan H.C., Cheng Y.C. (2007). Ozone exposure in the culture medium inhibits enterovirus 71 virus replication and modulates cytokine production in rhabdomyosarcoma cells. Antivir. Res..

[47] Lin Y.C., Wu S.C. (2006). Effects of ozone exposure on inactivation of intra- and extracellular enterovirus 71. Antivir. Res..

[48] Xie S.H., Wu Y.S., Liu X.J., Fu Y.B., Li S.S., Ma H.W., Zou F., Cheng J.Q. (2016). Mortality from road traffic accidents in a rapidly urbanizing Chinese city: A 20-year analysis in Shenzhen, 1994–2013. Traffic Inj. Prev..

[49] Beamish L.A., Osornio-Vargas A.R., Wine E. (2011). Air pollution: An environmental factor contributing to intestinal disease. J. Crohn’s Colitis.

[50] Tian L., Qiu H., Sun S., Tsang H., Chan K.P., Leung W.K. (2017). Association between emergency admission for peptic ulcer bleeding and air pollution: A case-crossover analysis in Hong Kong’s elderly population. Lancet Planet. Health.

[51] Ciencewicki J., Jaspers I. (2007). Air pollution and respiratory viral infection. Inhal. Toxicol..

[52] Gerba C.P., Rose J.B., Haas C.N. (1996). Sensitive populations: Who is at the greatest risk?. Int. J. Food Microbiol..

[53] Bouman A., Heineman M.J., Faas M.M. (2005). Sex hormones and the immune response in humans. Hum. Reprod. Update.

