# Supplementary material for: Short-Term Effects of Meteorological Factors and Air Pollutants on Hand, Foot and Mouth Disease among Children in Shenzhen, China, 2009–2017
Source: Int J Environ Res Public Health. 2019 Sep 27;16(19):3639. doi: 10.3390/ijerph16193639 (PMC6801881; doi:10.3390/ijerph16193639)
Supplement: Supplementary file 1 [file ijerph-16-03639-s001.pdf]

International Journal of
Environmental Research and Public Health

MDPI

Supplemental Material

Short-term effects of meteorological factors and air pollutants on hand, foot and mouth disease among children in Shenzhen, China, 2009–2017

Siyu Yan, Lan Wei, Yanran Duan, Hongyan Li, Yi Liao, Qiuying Lv, Fang Zhu, Zhihui Wang, Wanrong Lu, Ping Yin, Jinquan Cheng and Hongwei Jiang

Contents

Evaluating indicators 2
Section 1Choices of the degree of freedom for exposure and lag in cross-basis function 2
Table S1. The df of exposure and lag determined in the cb function. 2
Section2 Controlling for long-term trends and seasonality 2
Table S2. The QAIC and sum of PACF for different methods. 3
Figure S1. QAIC and sum of PACF for different *df* of time splines. 3
Section3 Controlling for autocorrelations caused by disease transmission 3
Table S3. The magnitude of the PACF plot for the first two lag days in different models. 3
Figure S2. The ACF and PACF plot of residuals after removing long-term trends and seasonality (by the time splines with 8 df per year). 4
Section 4 Results of univariate models 4
Figure S3. The estimated overall cumulative association between meteorological variables and HFMD occurrence over 14 days with their distributions, using a natural cubic spline DLNM in uni-meteorological variable models. The red lines are the cumulative relative risks (medians as references), and the gray regions are 95% CIs. Shenzhen 2009-2017. 5
Section 5 Results of multivariate models 5
Table S4. Spearman's correlation coefficients between meteorological variables and air pollutants in Shenzhen, China, 2009–2017. 5
Table S5.QAIC of multi-meteorological factor models 5
Table S6. Compare of the different functions of covariates. 6
Section 6 Sensitivity analysis 7
Figure S4. Results of sensitivity analyses by changing the df for controlling long-term trends and seasonality from 7 to 9 and changing the maximum lag days to 21 days, showing the estimated overall cumulative effects over maxlag days (except for NO_2 over lag0). 7
Figure S5. Lag-response curves with a max lag of 30 for P5, P95 of air temperature and relative humidity on HFMD occurrence. The red points are the relative risks (medians as references), and the black bars are 95% CIs. Shenzhen 2009-2017. 8
References 8

Evaluating indicators

Quasi Akaike Information Criterion (QAIC): QAIC is modified version of AIC to deal with the over-dispersed Poisson model, which can be used to assess the model fit of the quasi-Poisson regression model. It considers both the statistical fitness of the model and the number of parameters fitted. Besides, Peng et al. discussed the performance of model selection criteria such as AIC, BIC and PACF in time series studies of air pollutants and death, and proved AIC to be a better choice[1].

The sum of partial autocorrelation coefficients (Sum of PACF): The sum of PACF is the sum of the absolute value of the partial autocorrelation function (PACF) of the residuals (we set 14 days as the maximum lag in this analysis). The overall sum of PACF was used to compare the autocorrelation of residuals between different model choices. A smaller number means the residuals are less autocorrelated.

Section 1Choices of the degree of freedom for exposure and lag in cross-basis function

In the cb functions at the basic model, we set a 14d maximum lag and use natural cubic splines function both for exposure and lag. We varied the df of exposure from 2 to 6 and df of lag from 2 to 4 in order to select the best df combination with the smallest QAIC. Here are the results.

Table S1. The df of exposure and lag determined in the cb function.

Variables	df(var)	df(lag)
Air pressure	4	4
Temperature	4	4
Relative Humidity	6	4
Rainfall	2	2
Wind speed	2	3
Sunshine duration	4	2
SO_2	4	2
NO_2	2	2
CO	2	2
O_3	4	2
PM_{10}	3	2
$PM_{2.5}$	2	2

Section2 Controlling for long-term trends and seasonality

The time-series distribution curves of daily HFMD cases, meteorological factors and air pollutants show long-term trends and seasonality. The seasonal and long-term patterns in both the exposure and outcome data can dominate crude associations, making the short-term associations of interest hard to detect. By explicitly controlling for long-term patterns, the effects of exposure variable(s) of interest and the short-term variation around these long-term patterns can be explored. Previous literatures suggested that we could use the following methods to control for the long-term trends and seasonality [2]:

Option1: Spline function of time

It can model long-term patterns smoothly and capture seasonal patterns in a way that is allowed to vary from one year to the next. However, a controversial issue is determining how much smoothness, measured with degrees of freedom (*df*) of spline function[1]. Too few will fail to capture the main long-term patterns closely, whereas too many will result in a very 'wobbly' function which may compete with the variable of interest to explain the short-term variation of interest, widening confidence intervals of relative risk estimates.

In our studies, we varied the df of time splines from 2 to 13 df per year to explore its impact. We evaluated the results of different dfs based on QAIC and the sum of PACF. The results were displayed in table S1 and Figure S1. From the curves we found that when the *df* equaled 8 per year, both the QAIC and sum of PACF declined dramatically and remained at a relatively stable level. The results

agreed with the previous literature [3]. Therefore, we set the *df* of time splines as 8 per year in our final model.

option2: Time stratified case-crossover design

We compared another method, the time-stratified case crossover design, to control for the unmeasured time-varying confounding. Compared with the method of spline function of time, this method was not suitable based on the results of the higher QAIC and sum of PACF.

Table S2. The QAIC and sum of PACF for different methods.

Index	Df of time splines												Cross-over Design
	2	3	4	5	6	7	8	9	10	11	12	13	
QAIC	140407	109951	92730	51547	51144	45834	39944	39635	38296	37168	35982	34842	70585
PACF	1.95	1.79	2.16	2.92	2.87	1.79	1.60	1.64	1.62	1.56	1.47	1.42	2.27

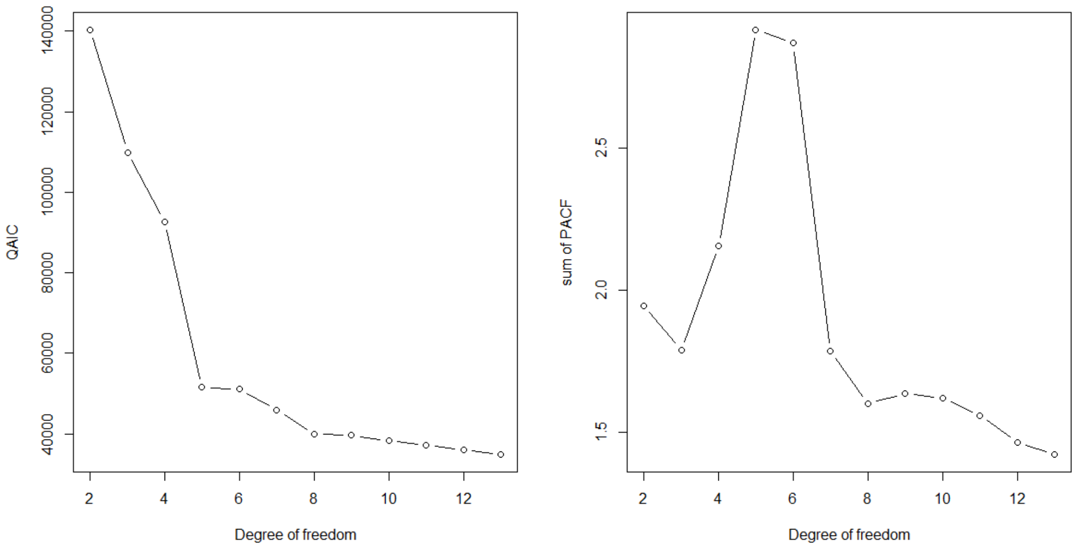

Figure S1. QAIC and sum of PACF for different *df* of time splines.

Section3 Controlling for autocorrelations caused by disease transmission

When the absolute magnitudes of the PACF plot for the first two lag days were both less than 0.1, the basic model was regarded as adequate; if this criterion was not met, autoregression terms for lag up to 7 days were introduced to improve the model. After our exploration, we found that model with autoregression terms for lag up to 2 days was adequate (showed in table 2 and Figure S2).

Table S3. The magnitude of the PACF plot for the first two lag days in different models.

Models	Lag=1	Lag=2
model without autoregressive terms	0.575	0.225
model with lag1 autoregressive term	-0.123	0.092
model with lag1 and lag2 autoregressive term	-0.019	-0.079

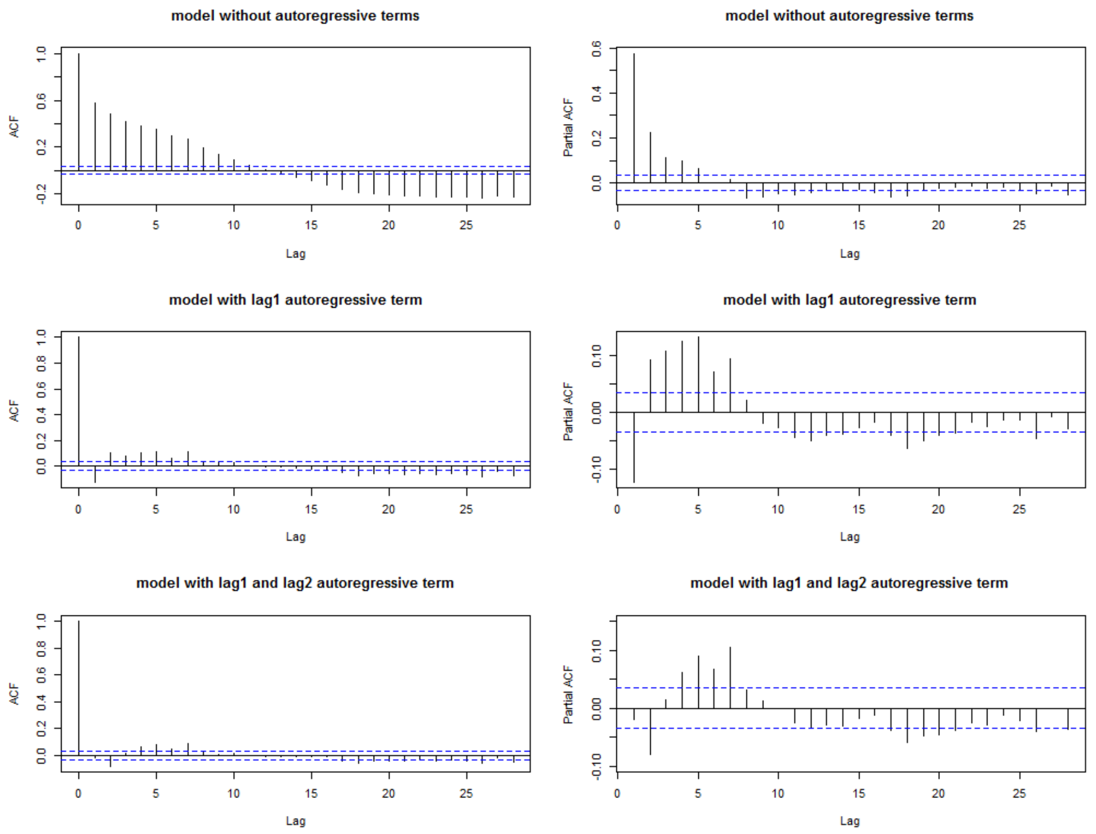

Figure S2. The ACF and PACF plot of residuals after removing long-term trends and seasonality (by the time splines with 8 df per year).

Section 4 Results of univariate models

In the univariate analysis, the overall cumulative effects of temperature, relative humidity, rainfall and sunshine duration were significant (Figure S3). The effect of temperature showed an obviously inverted V-shape and the cumulative RR value reached maximum 1.33 (95%CI: 1.24，1.42) at 28.7°C. However, the cumulative effect of air pressure and wind speed appeared to be non-significant on HFMD incidence.

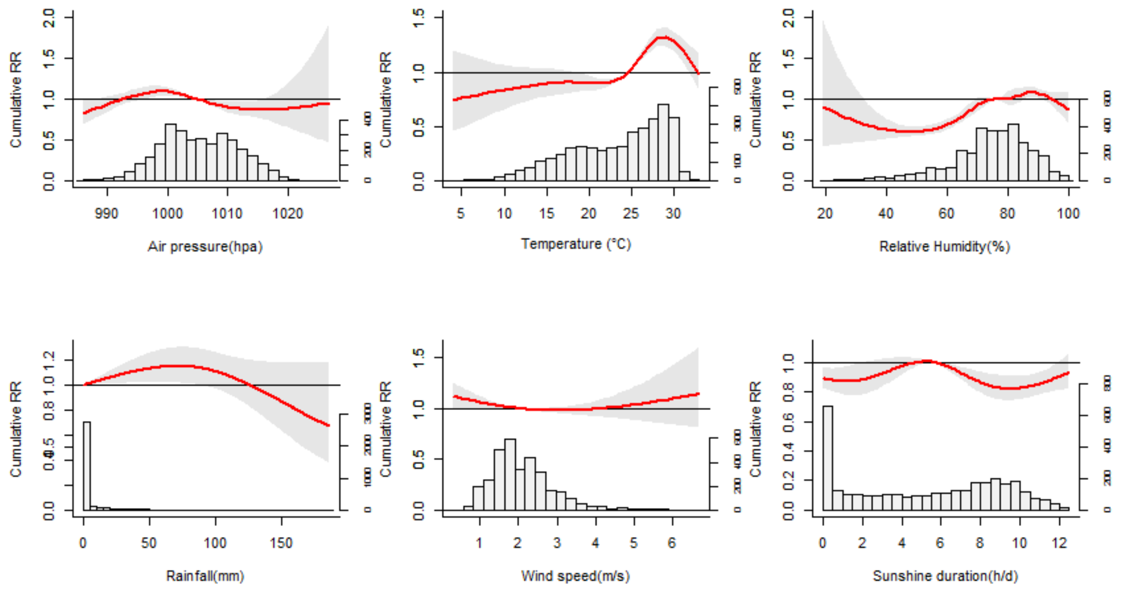

Figure S3. The estimated overall cumulative association between meteorological variables and HFMD occurrence over 14 days with their distributions, using a natural cubic spline DLNM in uni-meteorological variable models. The red lines are the cumulative relative risks (medians as references), and the gray regions are 95% CIs. Shenzhen 2009-2017.

Section 5 Results of multivariate models

Table S4. Spearman's correlation coefficients between meteorological variables and air pollutants in Shenzhen, China, 2009–2017.

Variables	Air pressure	Temperature	Relative Humidity	Rainfall	Wind	Sunshine	SO_2	NO_2	CO	O_3	PM_{10}
Temperature	-0.85^{**}										
Relative Humidity	-0.42^{**}	0.19^{**}									
Rainfall	-0.32^{**}	0.09^{**}	0.60^{**}								
Wind	0.07^{**}	-0.13^{**}	-0.16^{**}	-0.05^{**}							
Sunshine	-0.13^{**}	0.42^{**}	-0.46^{**}	-0.46^{**}	-0.01						
SO_2	0.24^{**}	-0.15^{**}	-0.50^{**}	-0.38^{**}	-0.07^{**}	0.25^{**}					
NO_2	0.30^{**}	-0.36^{**}	-0.14^{**}	-0.11^{**}	-0.25^{**}	-0.11^{**}	0.61^{**}				
CO	0.28^{**}	-0.34^{**}	-0.18^{**}	-0.06^{**}	0.07^{**}	-0.17^{**}	0.33^{**}	0.42^{**}			
$O_{3(2009\text{-}2016)}$	0.19^{**}	-0.08^{**}	-0.52^{**}	-0.36^{**}	0.08^{**}	0.22^{**}	0.30^{**}	0.09^{**}	0.07^{**}		
PM_{10}	0.47^{**}	-0.39^{**}	-0.53^{**}	-0.42^{**}	-0.08^{**}	0.07^{**}	0.66^{**}	0.63^{**}	0.38^{**}	0.57^{**}	
$PM_{2.5(2013\text{-}2017)}$	0.56^{**}	-0.51^{**}	-0.50^{**}	-0.41^{**}	-0.07^{**}	-0.02	0.57^{**}	0.59^{**}	0.54^{**}	0.33^{**}	0.96^{**}

$^{**}P<0.01$.

Table S5. QAIC of multi-meteorological factor models

Models	QAIC
AT+RH+SS+RF	31173
AT+RH+SS	31171
AT+RH+RF	31177
AT+SS+RF	31725
RH+SS+RF	31466
**AT+RH**	**31169**
AT+RF	31829
AT+SS	31712
RH+RF	31579
RH+SS	31451
RF+SS	32385
RF	32407
RH	31591
SS	32397
AT	31897

Abbreviations: AT, air temperature; RH, relative humidity; RF, rainfall; SS, sunshine duration.

We developed multivariate models to control the influence of other meteorological factors. The first thing was to choose the appropriate function for the meteorological covariate. Considering the non-linear and lagged effect of meteorological factors reported in previous literature, here are some choices.

In consideration of the non-linearity:

1. Natural cubic spline (ns) of the current value, which was commonly used in previous studies. But this method didn't consider about the lag effect of these meteorological variables, which may be non-ignorable according to our previous results. So we considered the following other methods;

In consideration of the lag effect:

2. The single lag value calculated by the function *Lag* in R package *tsModel*. But this method can only consider the single lag day effect at one time;
3. Moving Average, equaling mean of values of lag0 to maximum lag day, calculated by the function *runMean* in R package *tsModel*;
4. Exponential Moving Average, equaling exponentially-weighted mean of values of lag0 to maximum lag day, calculated by the function *EMA* in R package *TTR*;

Considering both the non-linearity and lag effect:

5. Combining the above functions, natural cubic spline of Moving Average/ Exponential Moving Average;
6. Generating a cross-basis matrix (cb) for the two dimensions of exposure and lags, calculated by the function *crossbasis* in package *dlnm*. The cross-basis matrix can distinguish effects of different lag days through user-specified functions and model the relationship of lag and response more elaborately.

We compared the above functions and evaluated the results based on QAIC, taking relative humidity and sunshine duration as examples. Temperature was included in each model for its main influence on HFMD supported from previous knowledge and literature. Both for relative humidity and sunshine duration, QAIC was the smallest when the cross-basis matrix of the variable was applied (Table S4). Therefore, we chose the cross-basis function for meteorological covariate finally.

Table S6. Compare of the different functions of covariates**.**

Relative humidity as covariate		Sunshine duration as covariate	
Model	QAIC	Model	QAIC
only cb(temperature)	31897	only cb(temperature)	31897
+ns(rh,df=3)	31908	+ns(sun,df=3)	31794
+Lag(rh,7)	31711	+Lag(sun,7)	31847
+MA(rh,14)	31428	+ MA (sun,14)	31743
+EMA(rh,14)	31554	+EMA(sun,14)	31837
+ns(MA (rh,14),df=3)	31431	+ns(MA (sun,14),df=3)	31757
+ns(EMA(rh,14),df=3)	31563	+ns(EMA(sun,14),df=3)	31853
+cb.rh (both ns)	31169	+cb.sun (both ns)	31712

Note: Rh= relative humidity;sun= sunshine duration;ns= Natural cubic spline function; MA= Moving Average; EMA= Exponential Moving Average.

Section 6 Sensitivity analysis

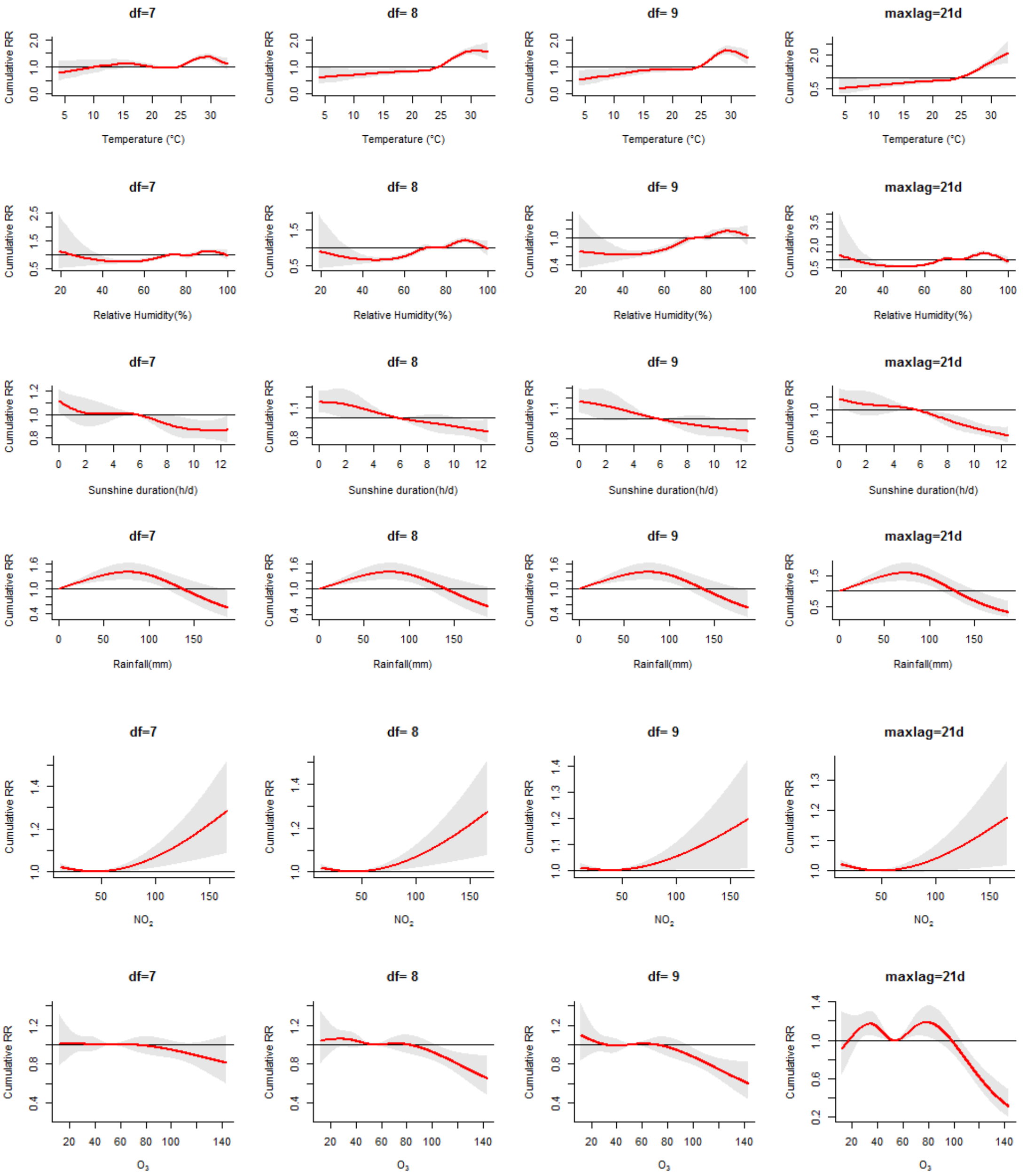

Figure S4. Results of sensitivity analyses by changing the df for controlling long-term trends and seasonality from 7 to 9 and changing the maximum lag days to 21 days, showing the estimated overall cumulative effects over maxlag days (except for NO_2 over lag0).

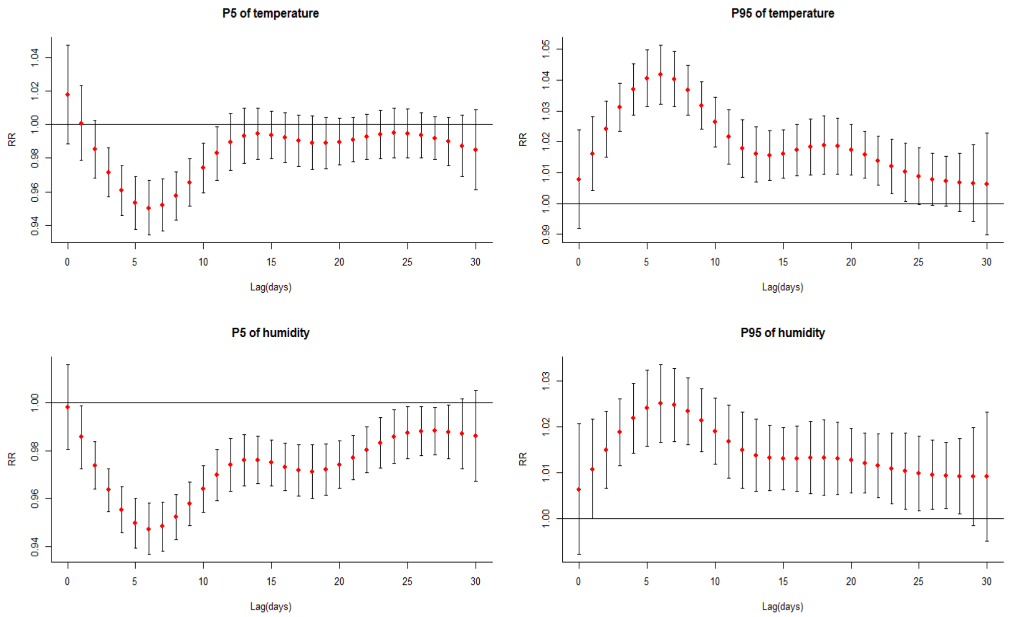

Figure 5. Lag-response curves with a max lag of 30 for P5, P95 of air temperature and relative humidity on HFMD occurrence. The red points are the relative risks (medians as references), and the black bars are 95% CIs. Shenzhen 2009-2017.

References

1. Peng, R. D., Dominici, F., Louis, T. A. Model choice in time series studies of air pollution and mortality. JOURNAL OF THE ROYAL STATISTICAL SOCIETY SERIES A-STATISTICS IN SOCIETY. 2006, 169(2): 179-198.
2. Bhaskaran, K., Gasparrini, A., Hajat, S., Smeeth, L., et al. Time series regression studies in environmental epidemiology. International Journal of Epidemiology. 2013, 42(4): 1187-1195.
3. Xiao, X., Gasparrini, A., Huang, J., Liao, Q., et al. The exposure-response relationship between temperature and childhood hand, foot and mouth disease: A multicity study from mainland China. Environment International. 2017, 100: 102-109.
4. Armstrong, B. Models for the relationship between ambient temperature and daily mortality. Epidemiology. 2006, 17(6): 624-631.
